# Momordica Grosvenori Shell-Derived Porous Carbon Materials for High-Efficiency Symmetric Supercapacitors

**DOI:** 10.3390/nano12234204

**Published:** 2022-11-26

**Authors:** Yunmeng You, Xianhao Hua, Yuanying Cui, Guiming Wu, Shujun Qiu, Yongpeng Xia, Yumei Luo, Fen Xu, Lixian Sun, Hailiang Chu

**Affiliations:** Guangxi Key Laboratory of Information Materials, Guangxi Collaborative Innovation Center of Structure and Property for New Energy and Materials, School of Materials Science and Engineering, Guilin University of Electronic Technology, Guilin 541004, China

**Keywords:** supercapacitors, Momordica grosvenori, porous carbon materials, KOH activation, electrode materials

## Abstract

Porous carbon materials derived from waste biomass have received broad interest in supercapacitor research due to their high specific surface area, good electrical conductivity, and excellent electrochemical performance. In this work, Momordica grosvenori shell-derived porous carbons (MGCs) were synthesized by high-temperature carbonization and subsequent activation by potassium hydroxide (KOH). As a supercapacitor electrode, the optimized MGCs-2 sample exhibits superior electrochemical performance. For example, a high specific capacitance of 367 F∙g^−1^ is achieved at 0.5 A∙g^−1^. Even at 20 A∙g^−1^, more than 260 F∙g^−1^ can be retained. Moreover, it also reveals favorable cycling stability (more than 96% of capacitance retention after 10,000 cycles at 5 A∙g^−1^). These results demonstrate that porous carbon materials derived from Momordica grosvenori shells are one of the most promising electrode candidate materials for practical use in the fields of electrochemical energy storage and conversion.

## 1. Introduction

The cumulative consumption of fossil fuels and the accompanying environmental pollution are two major global issues nowadays [1]. Reducing the growing dependence on fossil fuels is an essential goal at present [2]. As one of the most promising energy storage techniques, supercapacitors (SCs) have attracted a tremendous amount of attention due to their outstanding electrochemical properties, such as long cycling life, high power density, and fast charging/discharging rate [3,4]. The electrode materials for supercapacitors play a decisive role in their electrochemical properties. Electric double-layer capacitors (EDLCs) are generally electrochemically inactive materials [5], which do not participate in electrochemical reactions during the charging and discharging process. On the contrary, they are electrochemically active for pseudo-capacitors [6]. So far, carbon-based materials are a major focus for EDLCs [7,8,9]. With an adjustable pore structure and a special microscopic atomic arrangement, more electrochemically active sites can be obtained by increasing the specific surface area of carbon-based materials [10]. However, the disadvantages of poor cycling stability and low energy density hamper their widespread use [11]. Therefore, it is necessary to develop electrode materials with excellent electrochemical properties [12].

Carbon materials such as biocarbon [13,14] and carbon nanotubes (CNTs) [15,16] have been confirmed as appropriate electrode materials that reveal outstanding electrochemical properties in supercapacitors. However, the prohibitive cost and the relatively complicated preparation process with environmentally unfriendly effects of these carbon materials such as graphene and CNTs seriously limit their practical application. Biomass-derived carbon materials are perceived as a very promising alternative thanks to their low cost and abundant sources [17]. Additionally, waste biomass as a raw material for electrode materials can enable the renewable and sustainable application of resources. Moreover, the unique microstructure of porous carbons derived from biomass with a high specific surface area is conducive to electrolyte penetration and ion diffusion [18]. Up to now, various preparation methods have been employed to convert renewable biological resources into porous carbons with excellent electrochemical properties [19]. This waste biomass mainly includes potato [20], cicada slough [21], black locust seed dregs [22], litchi shell [23], bagasse [24], ginkgo leaves [25], and so on. Simultaneously, after the nitrogen atoms are doped into the carbon atomic lattice, they can effectively enhance the bonding of ions at the surface of carbon materials. Additionally, the specific capacitance of carbon materials can be greatly improved due to the generation of pseudo-capacitance resulting from N-doping in porous carbons. In addition, when nitrogen heteroatoms are doped into the graphite lattice, the electric conductivity of carbon materials is distinctly enhanced. Kumar et al. reported a tubular carbon with a porous structure (942 m^2^·g^−1^) extracted from the fruit of American poplar, and a superior specific capacitance (423 F·g^−1^ at 1 A·g^−1^) and rate capability (217 F·g^−1^ at 200 A·g^−1^) are achieved as electrodes in supercapacitors. Note that the capacitance decay is only 3% after 200,000 cycles at 200 A·g^−1^ [26]. Nevertheless, there is still a puzzle of low energy density, which seriously limits the widespread application of supercapacitor devices [27]. Therefore, increasing the energy density is particularly critical. In addition, tuning the microstructure of porous carbons also plays a crucial role in improving their electrochemical properties [28].

In this work, we adopted high-temperature carbonization and subsequent KOH activation to prepare porous carbons (MGCs) derived from Momordica grosvenori shells. Due to the unique morphological structure of Momordica grosvenori shells, MGCs exhibit excellent electrochemical properties as electrode materials for supercapacitors. The direct production of porous carbons with a promising electrochemical performance from waste biomass offers a hopeful perspective for energy conversion and storage.

## 2. Materials and Methods

### 2.1. Chemical Reagents

Momordica grosvenori used in this work was purchased from Guilin, Guangxi, China. Potassium hydroxide (KOH, 99.9%) and hydrochloric acid (HCl, 37%) were provided by Xilong Chemical Co., Ltd. (Shantou, China). Nickel foam (99.5%) with a thickness of 2 mm was provided by Lizhiyuan (LZY) Battery Material Co., Ltd. (Taiyuan, China).

### 2.2. Preparation of Porous Carbon Materials

The synthesis process of N-doped porous carbon is shown in Figure S1. Firstly, the Momordica grosvenori shells were washed with deionized water. After heating at 100 °C for 12 h, they were pulverized. The obtained powder was put into a tube furnace in a nitrogen atmosphere for annealing at 350 °C at 3 °C·min^−1^ and kept for 2 h to obtain the pre-carbonized materials that are denoted as PC-MGCs. Subsequently, 1 g of PC-MGCs was mixed with 2.5 g of KOH and ground uniformly. The resultant mixture was heated at 800 °C at 5 °C·min^−1^ and kept for 2 h under a nitrogen atmosphere. Finally, the solid product was immersed in 1 M HCl solution for 12 h to remove excess alkali and inorganic salts. The resultant product was repeatedly washed using deionized water by centrifugation and then heated at 80 °C to obtain MGCs-2. For comparison, several control samples were also synthesized under the same conditions except for changing the amount of KOH (0, 1.5, and 3.5 g), which were named as MGCs-0, MGCs-1, and MGCs-3, respectively. The mass ratio of carbon to KOH is 1:0, 1:1.95, 1:3.25, and 1:4.55 for all four samples.

### 2.3. Characterization

The microstructures and morphologies of MGCs were characterized by Quanta 450 FEG scanning electron microscopy (SEM, FEI, Hillsboro, OR, USA) and JEM-1200EX transmission electron microscopy (TEM, JEOL Ltd., Tokyo, Japan). The structures of MGCs were further studied with Raman spectroscopy (LabRAM HR Evolution, Horiba JY, Palaiseau, France) with a 532 nm laser beam and X-ray diffraction (XRD, Bruker D8 Advance, Karlsruhe, German) with Cu–Kα radiation. The nitrogen adsorption–desorption isotherms of the samples were obtained on a Quadrachrome adsorption instrument (ASIQM0002-4, Boynton Beach, FL, USA) at 77 K to determine the pore size distribution through Barrett-Joyner-Halenda (BJH) analysis and the total specific surface area (*S*_BET_) by the Brunauer-Emmett-Teller (BET) method. The surface elemental states of MGCs were obtained by X-ray photoelectron spectroscopy (XPS, Thermo Scientific Escalab 250Xi, Waltham, MA, USA).

### 2.4. Electrochemical Measurements

The electrochemical properties of MGCs as supercapacitor electrode materials were measured on an electrochemical workstation (Chenhua CHI660E, Shanghai, China). The working electrode was fabricated by mixing MGC active material, polytetrafluoroethylene (PTFE), and acetylene black at 8:1:1 (mass ratio). After adding a few drops of absolute ethanol, these materials were ground to obtain a uniform mixture, which was pressed on nickel foam (2 × 4 cm^2^) under a pressure of 8 MPa for 40 s. The active material in the as-prepared electrode is 6.440 mg. Galvanostatic charge–discharge (GCD) and cyclic voltammetry (CV) were conducted to evaluate the electrochemical properties. The CV curves were measured over a voltage range of −1.0 to 0 V at various scanning rates between 5 and 100 mV·s^−1^. GCD tests were performed in a three-electrode system in 6 M KOH aqueous solution with Pt wire as the counter electrode and Hg/HgO as the reference electrode. The determination of the specific capacitance was based on GCD curves according to Equation (1).

(1) $$C_{S}=\frac{I\Delta t}{m\Delta V}$$

where *I* (A) is the discharging current, Δ*t* (s) is the discharging time, *m* (g) is the mass of active material in each electrode, and Δ*V* (V) is the discharge potential window in GCD curves.

The symmetric supercapacitors were fabricated with two identical MGCs-2 electrodes. The electrode is identical to that mentioned above. The electrochemical performance was measured in a two-electrode system in 6 M KOH aqueous solution. The energy density (*E*, Wh·kg^−1^), specific capacitance (*C_s_*, F·g^−1^), and power density (*P*, W·kg^−1^) were calculated by the following Equations (2)–(4).

(2) $$C_{S}=\frac{I\Delta t}{m\Delta V}$$

(3) $$E=\frac{C_{S}{\left(\Delta V\right)}^{2}}{2\times 3.6}$$

(4) $$P=\frac{E}{\Delta t}\times 3600$$

where *I* (A) and Δ*t* (s) are identical to those in Equation (1), *m* (g) is the average mass of active material on both electrodes, and Δ*V* (V) is the voltage window for charging and discharging subtracting IR drop during the discharging process.

## 3. Results and Discussion

KOH plays a vital role during the formation process of porous carbons. In this work, we adopted the grinding method to achieve a close contact between the sample and KOH, which can fully activate porous carbon materials with a larger specific surface area. KOH is decomposed through the reaction with carbons at high temperatures (Equation (5)). When the temperature is over 700 °C, the generated gas from the decomposition of K_2_CO_3_ (Equation (6)) can further expand the specific surface area of the samples. The other probable reactions may be expressed in the following Equations (7)–(9). According to the TG results, the calcination temperature is determined to be 800 °C because there is no significant weight loss of PC-MGCs (Figure S2a). In addition, the as-prepared MGCs-2 sample can retain 80% of its weight after 1200 °C (Figure S2b), indicating good thermal stability. In addition, the content of C, N, and O in the PC-MGCs precursor was determined to be 76.90, 5.33, and 17.78 wt.%, respectively, by EDS elemental analysis (Figure S3).

(5) $$6\mathrm{KOH}+2C\to {2K+2K}_{2}{\mathrm{CO}}_{3}{+3H}_{2}$$

(6) $$K_{2}{\mathrm{CO}}_{3}\to K_{2}{O+\mathrm{CO}}_{2}$$

(7) $$K_{2}O+C\to 2K+\mathrm{CO}$$

(8) $${\mathrm{CO}}_{2}+C\to 2\mathrm{CO}$$

(9) $$K_{2}{\mathrm{CO}}_{3}+2C\to 2K+3\mathrm{CO}$$

**Figure 1:**
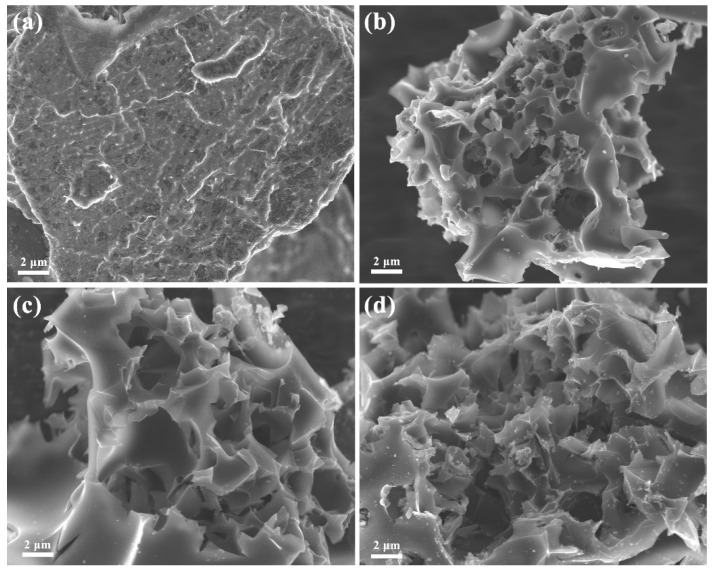
SEM images: (**a**) MGCs-0; (**b**) MGCs-1; (**c**) MGCs-2; (**d**) MGCs-3.

The SEM image in Figure 1a shows that the surface of MGCs-0 is relatively smooth without an apparent porosity structure due to the absence of KOH activation, which is not conducive to the transport of electrolyte ions and thus results in poor electrochemical performance. When we introduced different amounts of KOH, the samples exhibited different porous structures after KOH corrosion (Figure 1b–d). Especially for MGCs-2, its surface is very rough with a porous three-dimensional network structure (Figure 1c), which could store a mass of electric charge and also enhance the contact area between the electrolyte solution and the active material. With a further increase in the KOH amount, the sample of MGCs-3 is excessively corroded, destroying many micropores and mesopores and even leading to a collapse of the porous structure (Figure 1d). The uniform distribution of C, N, and O elements in the activated samples by the different amounts of KOH is confirmed by EDS elemental mappings (Figure S4). Their contents are provided in Table S1. With the increase in the KOH mass, the elemental amount of C is increased while the elemental amount of O is decreased. For the N element, there is a minimum for its amount for MGCs-2.

**Figure 2:**
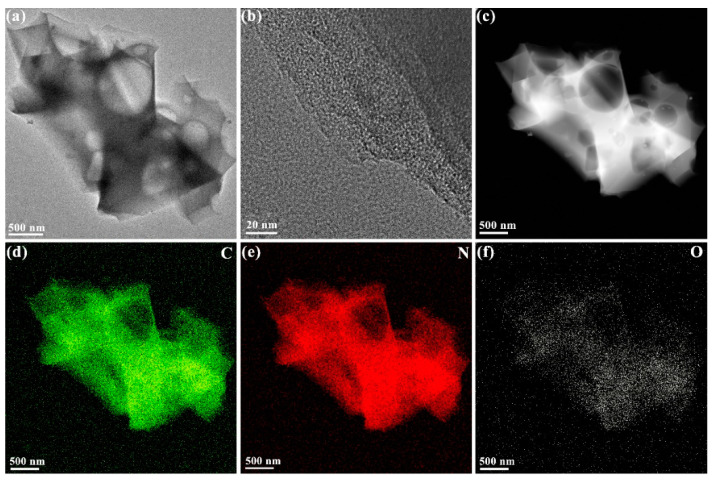
(**a**,**b**) TEM images and (**c**–**f**) EDX mapping of MGCs-2.

Moreover, plenty of pores throughout the carbon matrix with a clear interconnected porous framework are distinctly observed in the TEM image of MGCs-2 (Figure 2a). The inner porous structure can effectively shorten the ion diffusion path. The rapid transfer of electrolyte ions and electrons in MGCs-2 would give rise to superior electrochemical performance [29]. An HRTEM image demonstrates an amorphous state of MGCs-2 without any crystalline lattice fringes (Figure 2b). Again, C, O, and N elements are uniformly distributed in MGCs-2 according to its EDS elemental mappings shown in Figure 2c–f and Figure S5.

**Figure 3:**
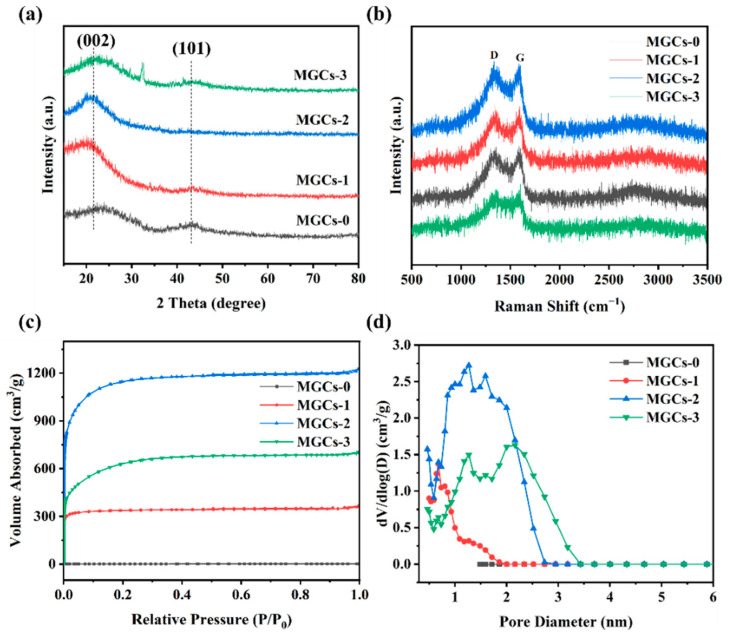
(**a**) XRD patterns; (**b**) Raman spectra; (**c**) nitrogen adsorption−desorption isotherms; (**d**) corresponding pore size distribution of MGCs.

The XRD patterns of the MGC samples are illustrated in Figure 3a. Two indistinct broad diffraction peaks are located at approximately 24° and 43°, which are due to the (002) and (101) crystal planes of graphite, also indicating the formation of typical amorphous carbon [30]. In Raman spectra (Figure 3b), two peaks at about 1580 and 1350 cm^−1^ are attributed to the G-band and D-band of carbon materials, respectively [31]. The D-band corresponds to structural defects associated with a disorganized carbon structure, whereas the G-band is related to the graphitic carbon [32]. The intensity ratio of *I_D_/I_G_* is commonly used to measure the degree of defects in carbon materials [31]. In all Raman spectra, the *I_D_/I_G_* value of each sample is greater than 1, indicating more defects and a lower degree of graphitization in these structures. The higher the ratio is, the more amorphous the structure is [33]. Among these four samples, MGCs-2 shows the highest *I_D_/I_G_* value of 1.076 (Table 1), indicating that it displays a structure with a higher disorder and lower graphitization, which is consistent with the XRD results. Additionally, the generation of more defects and disordered structures of MGCs-2 gives rise to an increase in the active surface area or active sites, which can enhance its specific capacitance. This conclusion is solidly confirmed by the following electrochemical GCD results.

It is well known that the energy storage principle for EDLCs is mostly *via* ion accumulation at the surface of the electrodes. Thus, *S*_BET_ is particularly important for carbon-based porous materials [34]. In general, a large *S*_BET_ of the electrode material can give rise to a large specific capacitance [35]. *S*_BET_ mainly depends on the size and number of pores in samples. The nitrogen adsorption–desorption isotherm was measured to determine the *S*_BET_ and pore size of the as-prepared MGCs samples (Table 1). As shown in Figure 3c, a characteristic I-type adsorption isotherm is observed for each MGC sample, which is the typical feature of microporous materials. In these isotherms, a sharp increase at *P/P*_0_ < 0.2 can be ascribed to the generation of abundant micropores. With a rise in the KOH content from 1.5 to 2.5 g, the porosity of the MGC samples becomes more evident. However, when the amount of KOH added is further increased to 3.5 g, it will result in the collapse of the porous structure (Figure 1d), and then the *S*_BET_ from the micropores will drop sharply (Table 1). According to nitrogen adsorption–desorption isotherms, MGCs-2 possesses a higher *S*_BET_ of 3996 m^2^·g^−1^ with a significant micropore content (72.3%) and a relatively low portion for mesopores (27.7%). Additionally, a very high micropore volume of 1.276 cm^3^·g^−1^ is determined for MGCs-2.

**Figure 4:**
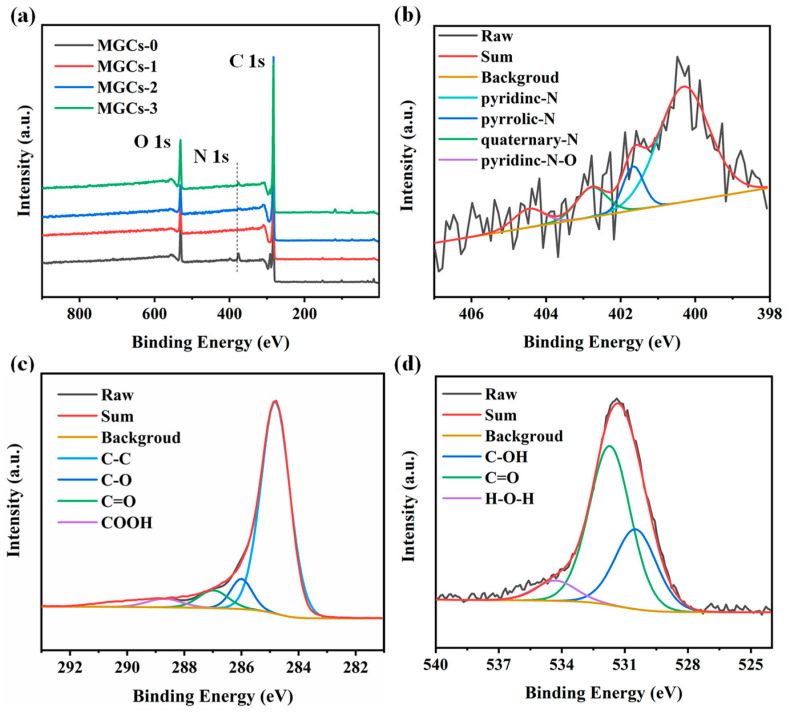
(**a**) Survey XPS spectra and high-resolution XPS spectra of (**b**) N 1s; (**c**) C 1s; and (**d**) O 1s of MGCs-2.

The chemical states of various elements of MGCs were explored by XPS analysis. It can be observed from the survey spectrum in Figure 4a that there are three binding energy peaks at about 284.8, 533.5, and 400.0 eV [36], reflecting the existence of C, O, and N. Through the deconvolution of the high-resolution N 1s spectrum, four peaks at 400.02, 401.26, 402.78, and 404.46 eV (Figure 4b) can be related to pyridinic-N, pyrrolic-N, quaternary-N, and oxidized-N [37], respectively. Thus, the content of pyrrolic-N in MGCs-2 is as high as 11.05%, which is considered electrochemically helpful for improving the electrochemical capacitance [38]. In addition, the high content of graphitic-N and quaternary-N in MGCs-2 can enhance its electrical conductivity [39]. The high-resolution XPS spectra of C 1s and O 1s are shown in Figure 4c,d, in which C 1s peaks appear at 288.70 eV (COOH), 286.98 eV (C=O), 286.10 eV (C-O), and 284.82 eV (C-C) [40], and O 1s peaks are at 530.15 eV (C-OH), 531.53 eV (C=O), and 534.2 eV (H-O-H) [41]. FT-IR spectroscopy results further confirm the existence of most of these C-containing and O-containing groups [42,43] (Figure S6). These functional groups of samples could promote their wettability, thereby enhancing the surface activity of the MGC samples [44].

**Figure 5:**
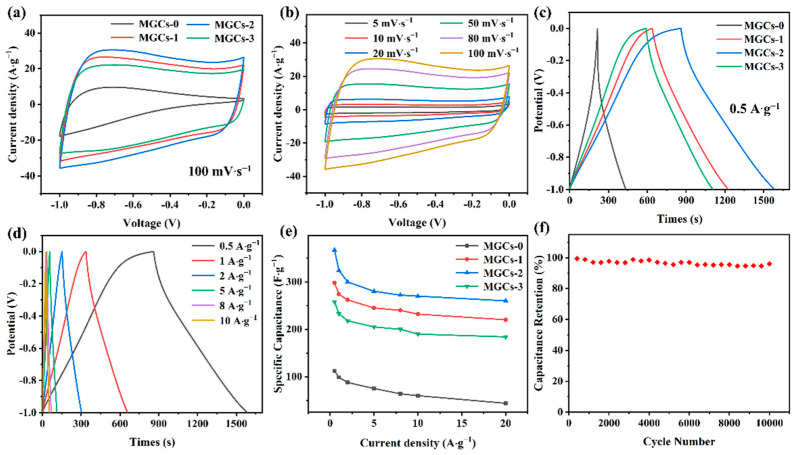
Electrochemical performance: (**a**) CV curves of MGCs at a scan rate of 100 mV∙s^−1^; (**b**) CV curves of MGCs-2 at different scan rates; (**c**) GCD curves of MGCs at a current density of 0.5 A∙g^−1^; (**d**) GCD curves of MGCs-2 at different current densities; (**e**) specific capacitances at various current densities of MGCs; (**f**) cycling stability at 5 A∙g^−1^ of MGCs-2.

The electrochemical performance of the as-synthesized MGCs samples as supercapacitor electrode materials was evaluated with a three-electrode system in a 6 M KOH solution. CV curves of MGCs were recorded at a scanning rate of 100 mV∙s^−1^ (Figure 5a). By comparison with MGCs-0, the CV curves of the KOH-activated samples (i.e., MGCs-1, MGCs-2, and MGCs-3) almost have a rectangular shape without redox-related peaks, implying typical EDLC behavior [45]. Among them, the CV of the MGCs-2 demonstrates the largest closed loop area, implying the highest specific capacitance. Moreover, the rectangular shape of the CV curves is almost maintained for MGCs-2 at various scanning rates (Figure 5b), implying fast charge and discharge characteristics. To obtain specific capacitance quantitatively, GCD tests were performed at 0.5 A∙g^−1^ (Figure 5c). An approximate symmetric triangle is observed for each GCD curve, which is indicative of the typical behavior of EDLCs. At 0.5 A∙g^−1^, the specific capacitance of the MGCs-2 is calculated to be 367 F∙g^−1^, much higher than the other samples (112 F∙g^−1^ for MGCs-0, 298 F∙g^−1^ for MGCs-1, and 258 F∙g^−1^ for MGCs-3). When the current density is increased to 1, 2, 5, 8, and 10 A∙g^−1^, its specific capacitance is as high as 324, 300, 280, 272, and 270 F∙g^−1^, respectively (Figure 5d). Even at 20 A∙g^−1^, a capacitance of 260 F∙g^−1^ is achieved with a capacitance retention rate of 70.8% (Figure 5e), indicating an outstanding high rate-performance of MGCs-2. Additionally, the stability of MGCs-2 was studied at 5 A∙g^−1^ for 10,000 consecutive cycles, a yielding high capacitance retention of 96% (Figure 5f). Table 2 shows that MGCs-2 exhibits better cycling stability than other biomass-derived carbon materials.

**Figure 6:**
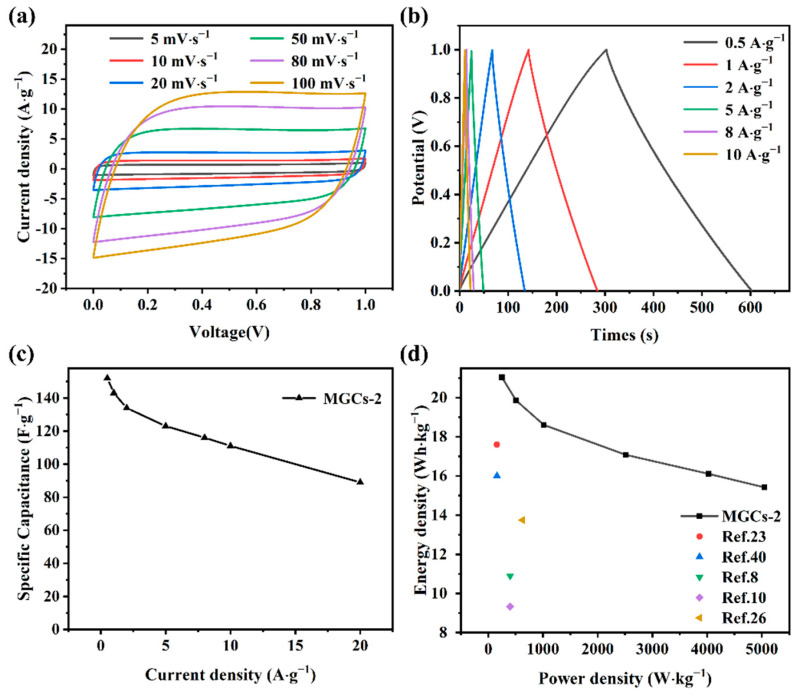
Electrochemical properties of SCD in 6 M KOH: (**a**) CV at various scanning rates; (**b**) GCD and (**c**) specific capacitance at different current densities; (**d**) Ragone plot.

As MGCs-2 shows superior capacitive performance, we assembled a two-electrode symmetric supercapacitor device (SCD) to deeply assess the electrochemical properties. A rectangular shape is nearly kept for each CV curve of SCD at different scanning rates (Figure 6a). From the GCD curves with a typical triangular-symmetric shape (Figure 6b), a high specific capacitance of 152 F∙g^−1^ is obtained for SCD at 0.5 A∙g^−1^, which may be due to the interconnected porous structure that can provide an unobstructed pathway for the efficient and fast transport/diffusion of electrolyte ions at high charge–discharge rates [51]. When the current density is further increased to 1, 2, 5, 8, and 10 A∙g^−1^, the specific capacitances are determined to be 143, 134, 123, 116, and 111 F∙g^−1^, respectively (Figure 6c). The capacitance retention is as high as 73% at 10 A∙g^−1^. From the Ragone plot (Figure 6d), a maximum energy density of 21.04 Wh·kg^−1^ is achieved for this SCD at a power density of 250 W·kg^−1^. At an energy density of 15.42 Wh·kg^−1^, the power density can reach 5047 W·kg^−1^. These results are comparable to those of previously reported carbon-based SCD with an alkaline medium (Figure 6d), demonstrating that the as-synthesized MGCs-2 sample is an economical and efficient electrode material for supercapacitors.

## 4. Conclusions

In summary, Momordica grosvenori shell-derived porous carbon materials were prepared through a two-step carbonization/KOH activation approach. The network porous structure endows the as-prepared MGCs-2 with high specific surface area (3996 m^2^∙g^−1^), superior specific capacitance (up to 367 F∙g^−1^ at 0.5 A∙g^−1^), better high-rate capability (260 F∙g^−1^ at 20 A∙g^−1^), and excellent cycling lifespan (96% retention rate after 10,000 cycles). More importantly, a high energy density of 21.04 Wh∙kg^−1^ is achieved for SCD that was assembled from MGCs-2. The electrochemical performance improvement can be related to the presence of N-containing functional groups such as pyridine nitrogen and the large microporous specific surface area of MGCs-2. N-containing species are beneficial to the enhancement of electrical conductivity and pseudo-capacitance. The inter-connected porous structure can promote the diffusion channels for electrolytes, thereby increasing the contact area between the electrolyte and the active material. These results suggest that the facile preparation of porous carbons derived from Momordica grosvenori shells is an economical and environmentally friendly strategy for advanced energy conversion and storage devices.

## Figures and Tables

**Table 1:** Microstructure of MGCs determined by nitrogen sorption isotherms and Raman spectra.

Sample	*S*_BET_ (m^2^·g^−1^)				Micropore Volume (cm^3^·g^−1^)	*I_D_/I_G_*
	Total	Micro	Meso	Ratio ^a^		
MGCs-0	1.07	0.57	0.50	1.14	0.0002	1.048
MGCs-1	1150	1042	108	9.64	0.473	1.047
MGCs-2	3996	2890	1105	2.61	1.276	1.076
MGCs-3	2251	947	1304	0.72	0.398	1.046

^a^ The ratio of specific surface area from micropores and mesopores.

**Table 2:** Comparison of the electrochemical properties of various reported biomass-derived carbon materials.

Precursor	Electrolyte	Current Density	Specific Capacitance	Capacitance Retention after 10,000 Cycles	Reference
Egg white	6 M KOH	0.5 A·g^−1^	335 F·g^−1^	80%	[46]
Ginkgo shell	6 M KOH	0.5 A·g^−1^	345 F·g^−1^	83%	[47]
Osmanthus fragrans	3 M KOH	0.5 A·g^−1^	351 F·g^−1^	93%	[48]
Cycas leaves	6 M KOH	0.5 A·g^−1^	373 F·g^−1^	85%	[49]
Fish seed	6 M KOH	0.5 A·g^−1^	350 F·g^−1^	94%	[50]
Momordica grosvenori shell	6 M KOH	0.5 A·g^−1^	367 F·g^−1^	96%	This work

## Data Availability

All data are available upon reasonable request.

## Supplementary Materials

The following supporting information can be downloaded at: https://www.mdpi.com/article/10.3390/nano12234204/s1; Figure S1: Schematic diagram of the synthesis of MGCs samples; Figure S2: TG curves of the as-prepared N-doped porous carbon; Figure S3: Energy dispersive spectrum of PC-MGCs; Figure S4: Energy dispersive spectrum of (a–d) MGCs-0; (e–h) MGCs-1; (i–l) MGCs-2; (m–p) MGCs-3; Figure S5: EDS mapping of MGCs-2; Figure S6: FT-IR spectra of the as-prepared N-doped porous carbon; Table S1: Elemental content of C, N, and O in the MGCs samples.
