# Supplementary material for: Momordica Grosvenori Shell-Derived Porous Carbon Materials for High-Efficiency Symmetric Supercapacitors
Source: Nanomaterials (Basel). 2022 Nov 26;12(23):4204. doi: 10.3390/nano12234204 (PMC9738515; doi:10.3390/nano12234204)
Supplement: Supplementary file 1 [file nanomaterials-12-04204-s001.zip › nanomaterials-1950940-supplementary.pdf]

nanomaterials

Supplementary Materials

Momordica Grosvenori Shell-Derived Porous Carbon Materials for High-Efficiency Symmetric Supercapacitors

Yunmeng You, Xianhao Hua, Yuanying Cui, Guiming Wu, Shujun Qiu *, Yongpeng Xia, Yumei Luo, Fen Xu, Lixian Sun * and Hailiang Chu *

Guangxi Key Laboratory of Information Materials, Guangxi Collaborative Innovation Center of Structure and Property for New Energy and Materials, and School of Materials Science and Engineering, Guilin University of Electronic Technology, Guilin 541004, China

* Correspondence: qiushujun@guet.edu.cn (S.Q.); sunlx@guet.edu.cn (L.S.); chuhailiang@guet.edu.cn (H.C.)

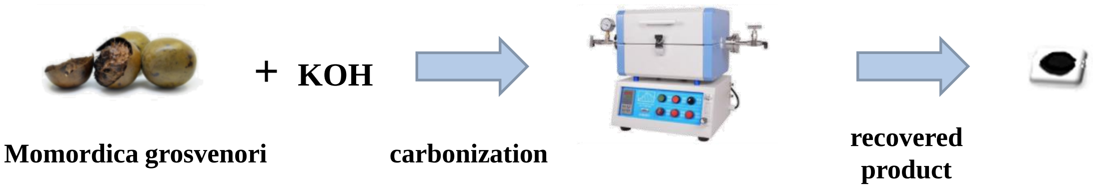

Figure S1. Schematic diagram of the synthesis of MGCs samples.

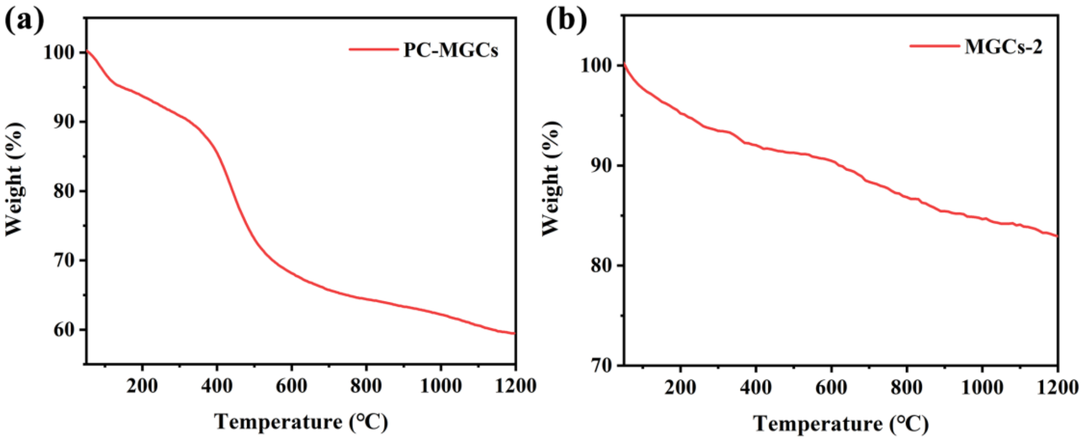

Figure S2. TG curves of the as-prepared N-doped porous carbon.

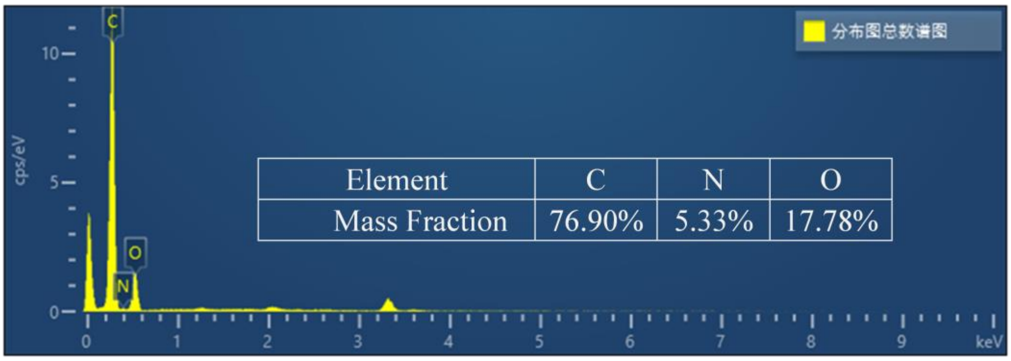

Figure S3. Energy dispersive spectrum of PC-MGCs.

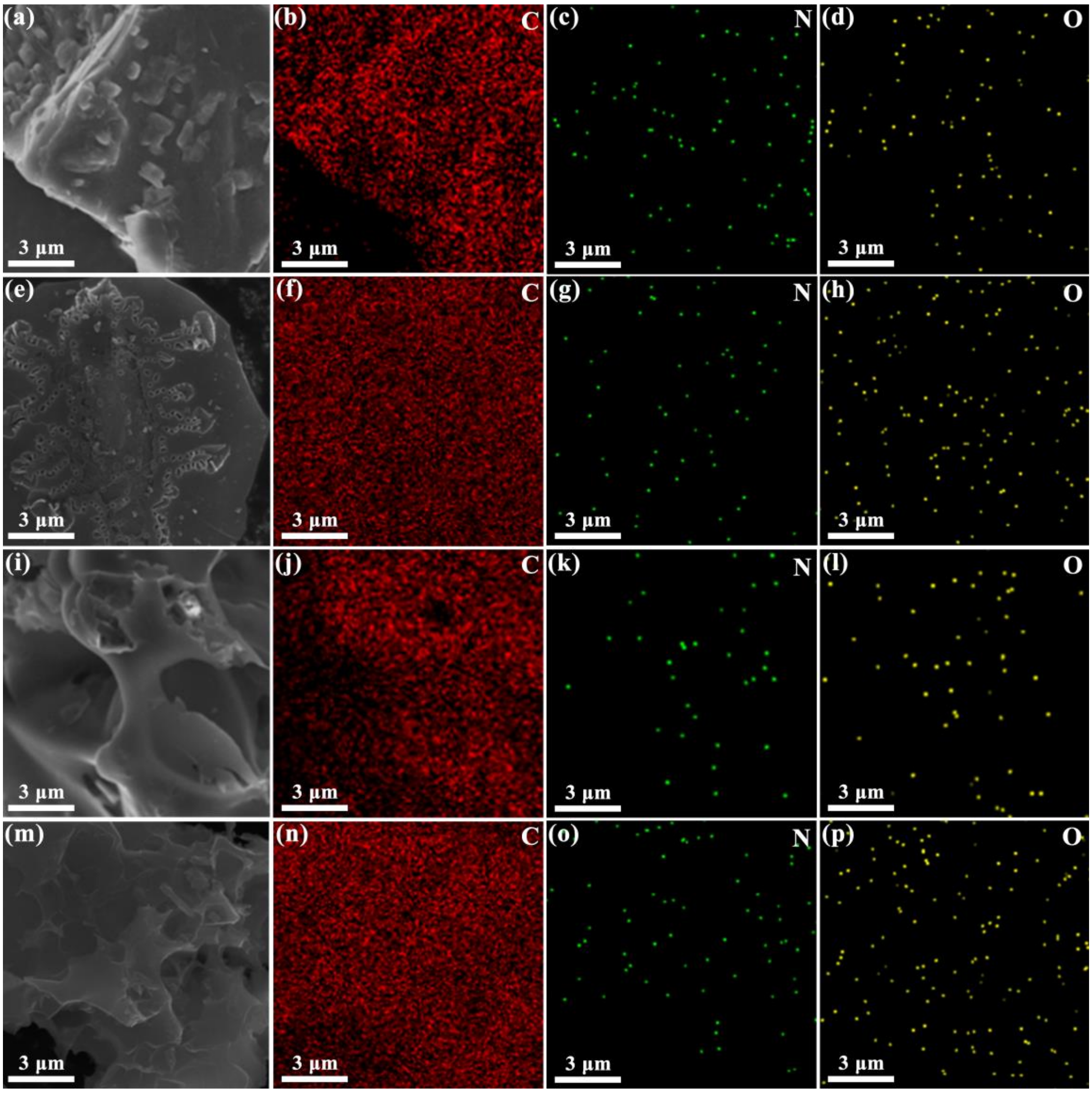

Figure S4. Energy dispersive spectrum of (**a**–**d**) MGCs-0; (**e**–**h**) MGCs-1; (**i**–**l**) MGCs-2; (**m**–**p**) MGCs-3.

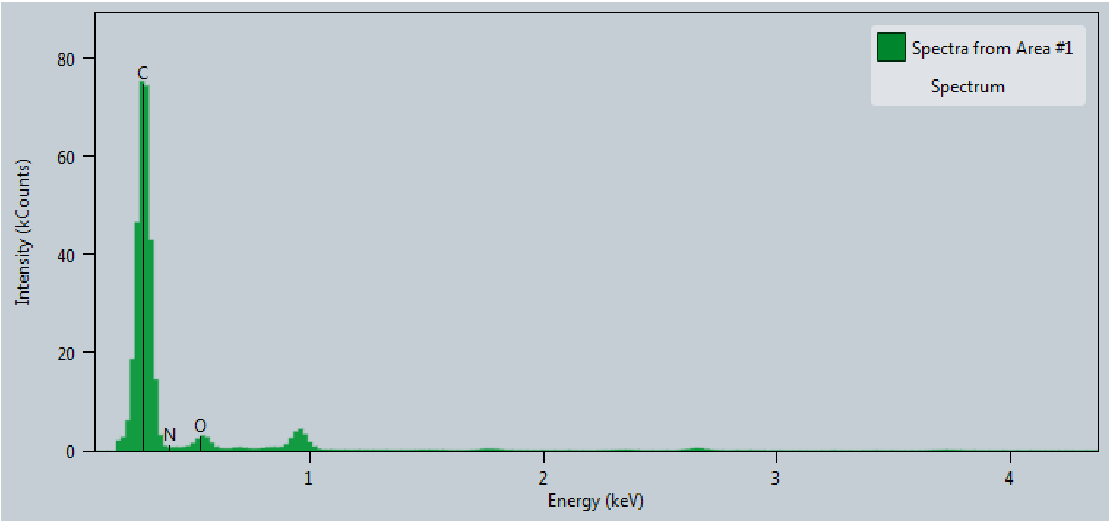

Figure S5. EDS mapping of MGCs-2.

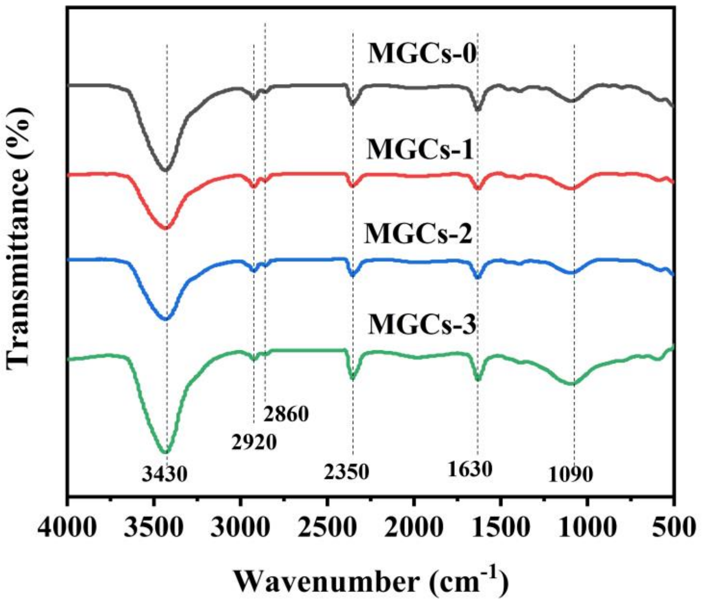

Figure S6. FT-IR spectra of the as-prepared N-doped porous carbon.

Table S1. Elemental content of C, N, and O in the MGCs samples.

Samples	C (wt.%)	N (wt.%)	O (wt.%)
MGCs-0	84.91	3.9	11.19
MGCs-1	87.12	2.13	10.76
MGCs-2	88.23	1.98	9.79
MGCs-3	88.29	2.17	9.54
